# miRNA deregulation and relationship with metabolic parameters after Mediterranean dietary intervention in BRCA-mutated women

**DOI:** 10.3389/fonc.2023.1147190

**Published:** 2023-04-04

**Authors:** Simona De Summa, Debora Traversa, Antonella Daniele, Orazio Palumbo, Massimo Carella, Raffaella Stallone, Antonio Tufaro, Andreina Oliverio, Eleonora Bruno, Maria Digennaro, Katia Danza, Patrizia Pasanisi, Stefania Tommasi

**Affiliations:** ^1^ Pharmacological and Molecular Diagnostics Unit, IRCCS Istituto Tumori Giovanni Paolo II, Bari, Italy; ^2^ Clinical Pathology Unit, IRCCS Istituto Tumori Giovanni Paolo II, Bari, Italy; ^3^ Division of Medical Genetics, Fondazione IRCCS Casa Sollievo Della Sofferenza, San Giovanni Rotondo, Foggia, Italy; ^4^ Biobank, IRCCS Istituto Tumori Giovanni Paolo II, Bari, Italy; ^5^ Department of Epidemiology and Data Science, Fondazione IRCCS Istituto Nazionale dei Tumori di Milano, Milan, Italy; ^6^ Department of Experimental Oncology IRCCS Istituto Nazionale dei Tumori di Milano, Milan, Italy; ^7^ Heredo-Familiar Cancer Clinic, IRCCS, Istituto Tumori Giovanni Paolo II, Bari, Italy; ^8^ Clinical Pathology Unit, “S. S. Annunziata” Hospital, Taranto, Italy

**Keywords:** miRNA, BRCA, mediterranean diet, dietary intervention, metabolism

## Abstract

**Background:**

Breast cancer onset is determined by a genetics-environment interaction. BRCA1/2 gene alterations are often genetically shared in familial context, but also food intake and hormonal assessment seem to influence the lifetime risk of developing this neoplasia. We previously showed the relationship between a six-months Mediterranean dietary intervention and insulin, glucose and estradiol levels in BRCA1/2 carrier subjects. The aim of the present study was to evidence the eventual influence of this dietary intervention on the relationship between circulating miRNA expression and metabolic parameters in presence of BRCA1/2 loss of function variants.

**Methods:**

Plasma samples of BRCA-women have been collected at the baseline and at the end of the dietary intervention. Moreover, subjects have been randomized in two groups: dietary intervention and placebo. miRNA profiling and subsequent ddPCR validation have been performed in all the subjects at both time points.

**Results:**

ddPCR analysis confirmed that five (miR-185-5p, miR-498, miR-3910, miR-4423 and miR-4445) of seven miRNAs, deregulated in the training cohort, were significantly up-regulated in subjects after dietary intervention compared with the baseline measurement. Interestingly, when we focused on variation of miRNA levels in the two timepoints, it could be observed that miR-4423, miR-4445 and miR-3910 expressions are positively correlated with variation in vitaminD level; whilst miR-185-5p difference in expression is related to HDL cholesterol variation.

**Conclusions:**

We highlighted the synergistic effect of a healthy lifestyle and epigenetic regulation in BC through the modulation of specific miRNAs. Different miRNAs have been reported involved in the tumor onset acting as tumor suppressors by targeting tumor-associated genes that are often downregulated.

## Introduction

Breast cancer (BC) represents one of the most common malignancies affecting women worldwide (1). Its lifetime risk development is associated with alterations in autosomal genes, including the well-known Breast Cancer gene 1 and 2, respectively *BRCA1* and *BRCA2* (2), which have a high penetrance in, the so-called, Hereditary Breast and Ovarian Cancer (HBOC) syndrome.

However, genetic predisposition is not the only factor determining tumor onset. It has been reported that additional factors could promote tumor development and influence the cancer microenvironment, such as environmental and unhealthy lifestyle factors (for example poor diet with weight-gain, alcohol intake, and smoking) (3–5).

The elevated levels of blood glucose, insulin, free estradiol, and inflammatory cytokines as a consequence of an increased quantity of metabolically active adipose tissue, seems to affect BC risk and survival (6). It has been demonstrated that these factors favor cell proliferation at the expense of apoptosis, and by modifying cellular lipid architecture, promote migration and metastasis.

This highlights the importance of paying particular attention to susceptible subjects’ habits before the eventual BC onset or after that due to the strict relationship between genetics and metabolic and hormonal assessment influenced by lifestyle (i.e. diet). It is reported that *BRCA1/2* genes could be modulated by several metabolic and hormonal factors (7, 8) and their loss of function variants can influence insulin levels by a different response to the dietary intervention (9).

For instance, obesity is associated with a higher risk of BC in women with a high-risk family history, often in association with high serum levels of insulin-like growth factor I (IGF-I), deriving from high energy intake, that increases BC penetrance and influences the prognosis. Many foods, in fact, could stimulate insulin production or release, such as milk, and this is closely related with higher IGF-I plasma levels (10).

BRCA and IGF-I are functionally related since the IGF-I pathway seems to regulate the BRCA1 expression level while wild-type BRCA1 strongly reduces the synthesis of IGF-I receptors.

Bordeleau et al. (2011) showed that women BRCA-mutated with BC frequently developed type-2 diabetes (11), probably because the mutation induces the loss of BRCA1 anti-tumor activity, leading to an over-activation of IGF-IR signaling pathway (12).

Type-2 diabetes, in fact, is due to insulin resistance and subsequent altered glucose metabolism (13).

Several studies demonstrated that a balanced diet could improve the prognosis and the overall survival of BC patients, combined with conventional therapies, limiting their side effects (3, 11, 14, 15).

The Mediterranean diet, providing a high consumption of legumes, fruits, vegetables, dried fruit and, olive oil at the expense of sweets and red meat intake, may have a primary prevention effect on this tumor type, by reducing some potential modulators of BRCA penetrance, including circulating microRNAs (miRNAs). These molecules are widely expressed and regulate several biological processes, among which the glucose homeostasis, playing a direct role, for example, in the control of insulin production, secretion, and signaling (16).

Previous studies demonstrated a deregulation of miRNAs expression in subjects that followed a dietary intervention, also after a short time, highlighting modulation of metabolic parameters (6, 17).

Therefore, miRNAs expression levels, also in body fluids [such as plasma and serum samples in which miRNAs can remain highly stable (18)], could be considered as potential new biomarker in BC early diagnosis and monitoring, although it is not fully understood how this imbalance occur in cells and how diet and miRNAs expression are related.

In the present study we aimed to analyze the plasma miRNA profile in a sample of women with BRCA mutations included in a six-month Mediterranean dietary intervention trial (MedDiet trial, NCT03066856) (19). In this trial, we demonstrated that a Mediterranean dietary intervention is feasible and effective in reducing some potential modulators of BRCA penetrance, such as body weight, waist circumference and IGF-I expression, in the intervention compared to the control group. Here, we focus on understanding if there is a variation in the regulation of metabolic and inflammatory pathways potentially linked to BRCA penetrance and IGF pathway, following the expression levels of selected miRNAs after a Mediterranean dietary intervention.

## Materials and methods

### Patients

The multicenter two-arm prospective randomized controlled trial on BRCA 1/2 mutant women has been fully previously described (19).

Briefly, that study investigated whether an active dietary intervention based on the ‘Mediterranean diet’ with moderate protein restriction significantly reduced IGF-I and other modulators of BRCA penetrance. Eligible study subjects were women, aged 18-70, with or without a previous diagnosis of BC/OC, without metastases, who underwent genetic counselling and fulfilled high-risk selection criteria for genetic testing, and were found to be carriers of deleterious *BRCA* mutations (**Supplementary Table 1** list the full alterations of the enrolled subjects).

Among the 416 volunteers, 216 randomized in the intervention group and 200 in the control group, who concluded the six-month dietary intervention, 56 have been recruited/randomized at the IRCCS – Istituto Tumori “Giovanni Paolo II” from September 2017 and July 2019 and included into the present miRNAs analysis. The study was approved by the local Ethics Committee (CE n. 597/2016), and all women gave their signed informed consent. At baseline and at the end of the six-month dietary intervention, all study participants underwent anthropometric and plicometric examinations and gave 20 ml of blood to measure all the hormonal and metabolic parameters under study (20, 21). The percentage of fat mass (FT) was carried out using a FAT-1 plicometer (GIMA- Italy) which measures the thickness of skin folds in various districts and assesses the nutritional status of the subject under examination and the sectoral distribution of its adipose tissue. The Durnin-Womerslay measurement of 7 folds (bicipital, tricipital, axillary, subscapular, abdominal, over-iliac and median thigh) was performed (22). Baseline features are displayed in **Table 1**. In **Supplementary Table 2**, the comparison of baseline features stratifying patients by sample set have been displayed.

**Table 1:** Baseline characteristics of the study population by treatment group.

Characteristic	Control, N=27^1^	Diet, N=29^1^	*p*-value^2^
Age	49 (38, 54)	48 (42, 55)	0.7
Height (cm)	163 (160,164)	162 (160, 165)	0.7
Fat Mass	34.7 (30.0, 40.1)	37.1 (32.5, 39.2)	0.5
Adiponectin (ng/ml)	9.3 (7.1, 13.8)	10 (5.8, 14.8)	0.7
Leptin (ng/ml)	20 (15, 4,876)	15 (11, 31)	0.065
Weight (kg)	60 (56, 70)	61 (54, 68)	0.8
BMI	22.9 (20.1, 26.9)	23.5 (21.1, 26.2)	0.7
Glycemia (mg/dl)	91 (87, 96)	89 (87, 92)	0.3
Cholesterol Tot (mg/dl)	196 (158, 218)	200 (175, 225)	0.4
Cholesterol HDL	63 (56,76)	69 (61, 81)	0.3
Cholesterol LDL	131 (90, 147)	132 (92, 157)	0.6
Triglycerides (mg/dl)	89 (71, 108)	72 (61, 91)	0.088
Vitamin D (μg/L)	28 (21, 34)	23 (18, 33)	0.3
IGF-I (ng/ml)	149 (121, 200)	132 (119, 172)	0.6
Insulin (μU/ml)	7 (4, 13)	6 (3, 7)	0.10
HOMA - IR	1.43 (0.89, 3.02)	1.24 (0.67, 1.46)	0.079
Gene mutation (%)			
*BRCA1*	14 (52)	20 (69)	0.2
*BRCA2*	13 (48)	9 (31)	

^1^n(%); Median (IQR).

^2^Wilcoxon rank sum test; Pearson’s Chi-squared test.

### Plasma collection and miRNAs isolation

Eight milliliters of peripheral blood were collected into Cell-Free DNA BCT (Streck Corporate, La Vista, NE) at each time-point and was centrifuged at 3000 rpm at 4°C for 15. The obtained plasma samples were further centrifuged at 16,000 g at 4°C for 10 min to remove cell debris. The separated plasma was stored at -80°C until nucleic acid extraction. Total RNA was isolated from plasma using *mir*Vana™ miRNA Isolation Kit (Ambion, Austin, TX, USA), according to the manufacturer’s recommendations.

### miRNA expression profiling

miRNA expression profiling was performed by using GeneChip™ miRNA 3.0 Array (Affymetrix, Santa Clara, CA) which contains 179,217 probes, representing 19,913 mature microRNA annotated in miRBase V.17, and covers 203 organisms of all species including human, mouse, and rat. For each sample, 500 ng of total RNA was labelled using the 3 DNA Array Detection Flash Tag RNA Labelling Kit (http://www.genisphere.com), according to the manufacturer’s instructions.

Firstly, poly (A) tailing was carried out at 37°C for 15 min in a volume of 15 ml reaction mix that contained 1 ml Reaction Buffer, 1.5 ml MgCl2 (25 mM), 1 ml ATP Mix diluted 1:500 and 1 ml PAP enzyme. Subsequently, Flash Tag Ligation was performed at room temperature for 30 min by adding 4 ml of 5 Flash Tag Ligation Mix Biotin and 2 ml T4 DNA Ligase into 15 ml of reaction mix. Next, 2.5 ml of Stop Solution was added to stop the reaction. Each sample was hybridized on the array, washed, stained with the Affymetrix Fluidics Station 450, and scanned with the Affymetrix Gene Chip Scanner 3000 7G using the Command Console software (Affymetrix).

### Array data processing and statistical analysis

Raw data were normalized with the Robust Multiarray Average (RMA) method to remove systematic variations. Briefly, RMA corrects raw data for background using a formula that is based on a normal distribution and uses a linear model to estimate values on a log-scale. The RMA normalization was performed using the ‘Affy’ R package. The normalized expression matrix has been filtered to retain only *hsa* probes, that is human miRNAs. Differentially expressed miRNAs were detected setting up a time-course analysis. In detail, differentially expressed miRNAs have been detected comparing the two time-points independently in the two groups. miRNAs were considered deregulated if logFC>|1.5| and adjusted p-value<0.05. The differential expression analysis has been performed with ‘*limma*’ R package. Then, through depicting a Venn diagram (http://jvenn.toulouse.inra.fr/app/example.html), miRNAs specifically deregulated in each of the two groups have been identified.

### Validation of selected miRNA expression with digital droplets PCR

The expressions of selected miRNAs from microarray were measured using ddPCR. Briefly, commercial Taqman probes of selected miRNAs were produced by ThermoFisher Scientific (Rodano MI, Italy). Specific reverse transcription of miRNA was performed using TaqMan™ MicroRNA Reverse Transcription Kit (ThermoFisher Scientific, Rodano MI, Italy). The preparation of ddPCR samples was performed according to the manufacturer’s protocol for ddPCR supermix for probes (Bio-Rad Laboratories, Inc., CA, USA). After the 96-well plate was loaded on and read by a QX200 Droplet Reader and the data were collected using QuantaSoftTM (Bio-Rad, Hercules, CA). U6 small nuclear RNA (snRNA) was selected as an internal normalizer RNA.

### Pathway enrichment analysis

To understand the biological role of miRNAs, the access to TarBase v8 (23) has been requested and granted. Such a database allowed the identification of the experimentally validated target genes of miRNAs. In detail, TarBase has been filtered to detect target genes that have direct interaction and down-regulation as an effect on expression. The list of selected genes was then used to perform pathway enrichment analysis, performed with the ‘*EnrichR*’ R package, including Gene Ontology (Biological Process, Molecular Function and Cellular Processes), KEGG and WikiPathways.

### Statistical analysis

To detect relationships with metabolic features, miRNA expression data coming from training and validation sets have been normalized and standardized with the ‘*caret*’ R package. Such a procedure allowed to merge the two sets and thus to gain statistical power.

A further metabolic parameter has been added to metabolic parameter, used to measure insulin resistance, HOMA - IR (Homeostatic Model Assessment for Insulin Resistance):


HOMA−IR =122.5(fasting serum insulin μUml X fasting plasma glucose mmoll)

Thus, miRNA expression data and metabolic features have been merged to create a dataset, including data coming collected at the two time-points. Delta values have calculated:


$$\Delta feature\;=\;feature_{last}\;-feature_{first}$$

where: *feature_last_* is the value detected at the second time point and *feature_first_* is the basal measure.

Pearson correlation analysis has been performed with R *cor()* function and statistical significance has been obtained with R *cor.mtest()* function. Finally, a correlation plot has been depicted with the *corrplot* R package.

Data, where necessary, have been categorized using median value as cut value. miRNA expression values have been compared with the Wilcoxon-Mann-Whitney test and boxplots were depicted with the *ggplot2* R package.

## Results

### miRNA expression profiling

**Figure 1:**
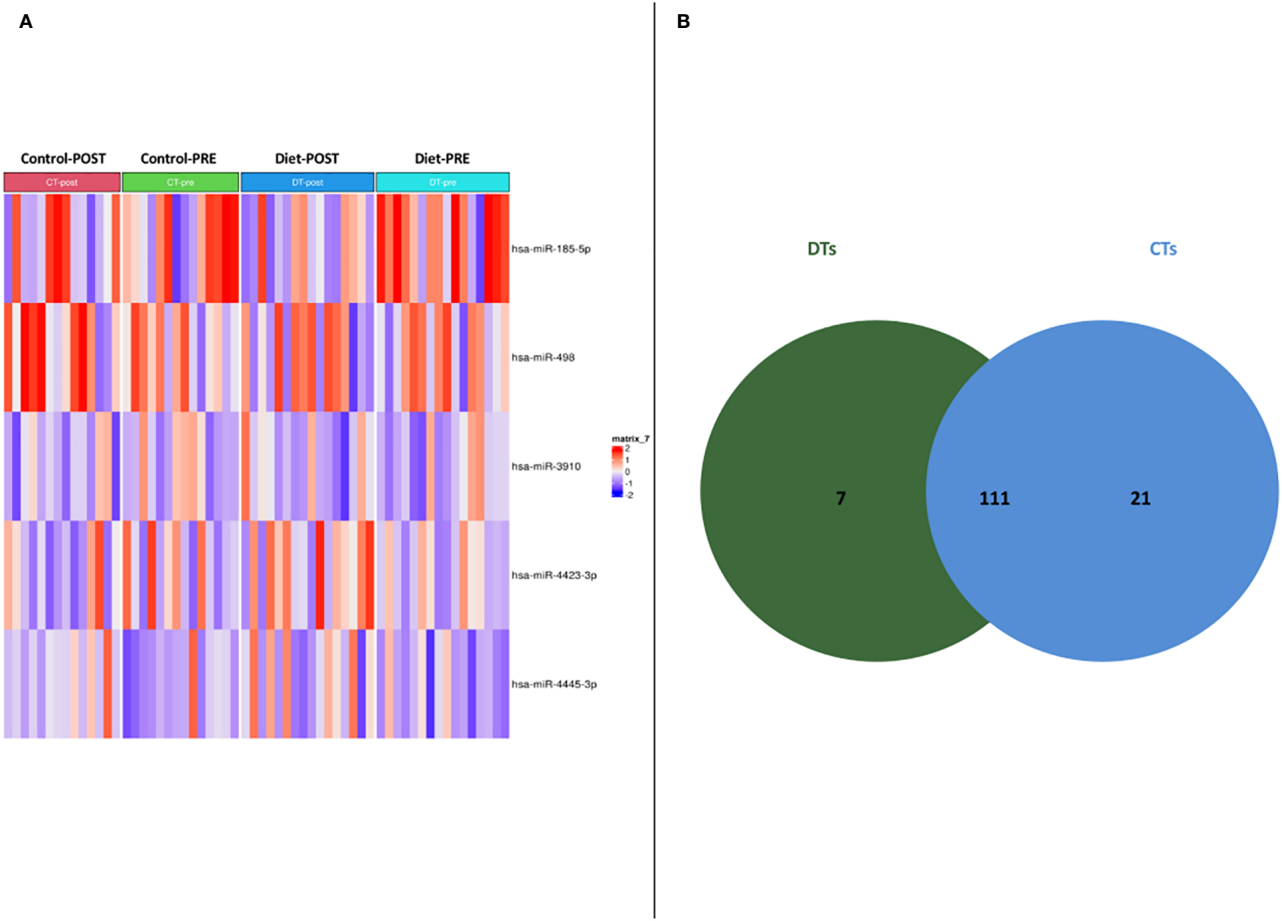
**(A)** Heatmap of DEmiRNAs in the two subgroups taking into account pre- and post- dietary intervention expression data. **(B)** Venn diagram depicting deregulated miRNA distribution in DTs and CTs.

56 women carriers of BRCA mutations entered in the present analysis and were splitted into training and validation sets. In detail, 30 women were included in the training cohort, including 16 patients that underwent dietary regimen and 14 control subjects (Dietary Training, DTs; Control Training, CTs, respectively); in the validation cohort, 26 patients out 13 underwent Mediterranean diet and 13 control cases (Dietary Validation, DV; Control Validation, CV, respectively). Both DTs and CTs underwent blood withdrawal at baseline and at the end of the six-month dietary intervention. miRNA expression profile was performed on training set by microarray analysis. Two-time course analyses have been performed both in DT and CT subgroups. Considering log2FoldChange > |1.5| and p-value<0.05, differential expression analyses, in time-course mode, highlighted 119 and 133 DEmiRNAs (Deregulated miRNAs) in DTs and CTs, respectively (**Figure 1A**). In order to identify DEmiRNAs specific for the DT subgroup, the two previous results were intersected. In **Figure 1B**, Venn diagram displays that 7 miRNAs are deregulated in the subgroup that underwent dietary intervention. In detail, miR-4423-3p, miR-185-5p, miR-4445-3p, miR-498, let-7d-5p and miR-1263 were exclusively upregulated in DTs (**Table 2**).

**Table 2:** DEmiRNAs exclusively detected in subjects of the DT subgroup.

	logFC	adjusted Pval
hsa-miR-4423-3p	1,888	4,47E-12
hsa-miR-185-5p	1,556	2,26E-08
hsa-miR-4445-3p	1,533	3,4E-09
hsa-miR-498	1,533	8,75E-16
hsa-let-7d-5p	1,519	1,38E-11
hsa-miR-1263	1,514	2,72E-15
hsa-miR-3910	1,507	7,34E-17

### DEmiRNA validation in independent subset

**Figure 2:**
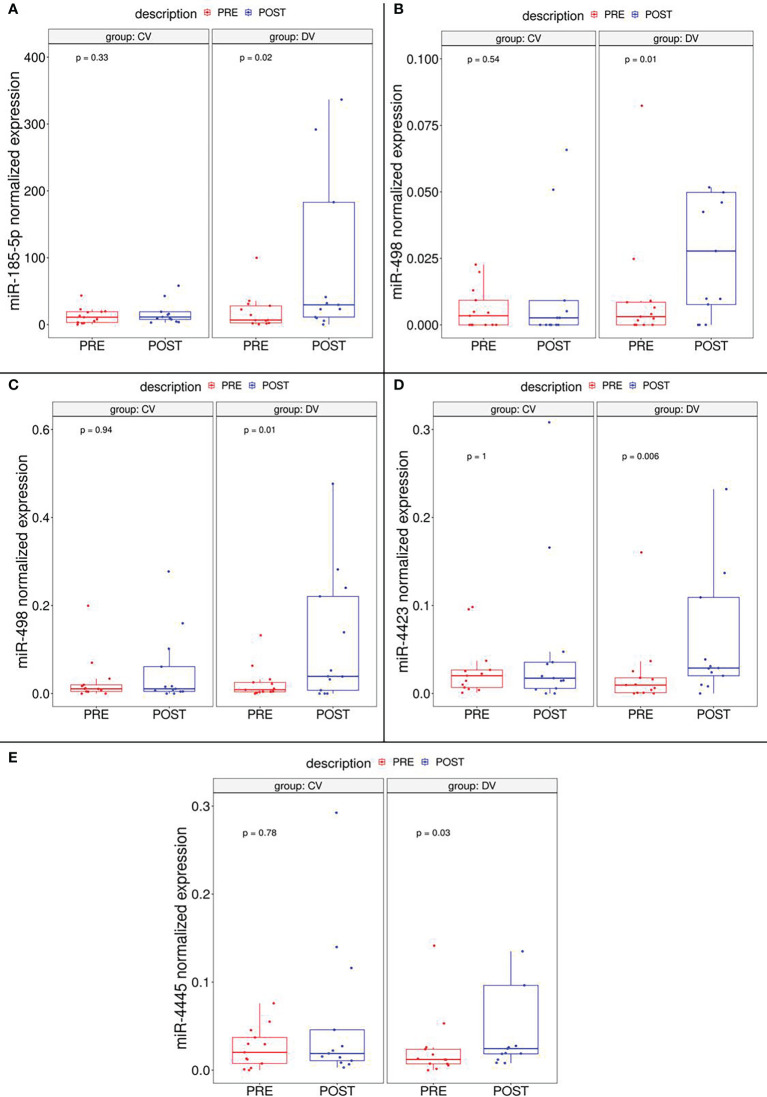
Boxplot depicting validated DEmiRNAs in independent cohort subgroups (DV and CV). It could be observed that miR-185-5p **(A)**, miR-498 **(B)** and miR-3910 **(C)**, miR-4423 **(D)** and miR-4445 **(E)** are significantly upregulated in DV subjects.

The validation cohort included 26 subjects randomized in the intervention or control group in a 50:50 proportion (DVs and CVs, respectively. Digital droplet PCR (ddPCR) was elected as validation method because of its high sensitivity, a specially needed parameter for the detection of circulating miRNAs. ddPCR analysis confirmed that five of seven miRNAs (miR-185-5p, miR-498, miR-3910, miR-4423 and, miR-4445) were significantly up-regulated in subjects after diet intervention compared with the baseline measurement before participation in the lifestyle modification (**Figure 2**). Two miRNAs (let-7d-5p and miR-1263) had no significant difference in expression levels between groups.

### Pathway enrichment analysis

To highlight how the validated DEmiRNAs act on cellular processes, and eventually exert metabolic influences, pathway enrichment analysis has been conducted. In detail, such analysis has been performed through experimentally validated target genes, as reported in the TarBase database.

The choired pathways enriched in the genes targeted by over-expressed miRNAs were associated with cellular proliferation and adhesion, cell-cycle, regulation of transcription, epithelial-mesenchymal transition (EMT), drug resistance, angiogenesis, chronic inflammation and diabetes.

The analysis also showed an enrichment in the phosphatidylinositol-3-kinase (PI3K)-protein kinase B (Akt) and AMP-activated protein kinase (AMPK) signaling pathway.

**Figure 3:**
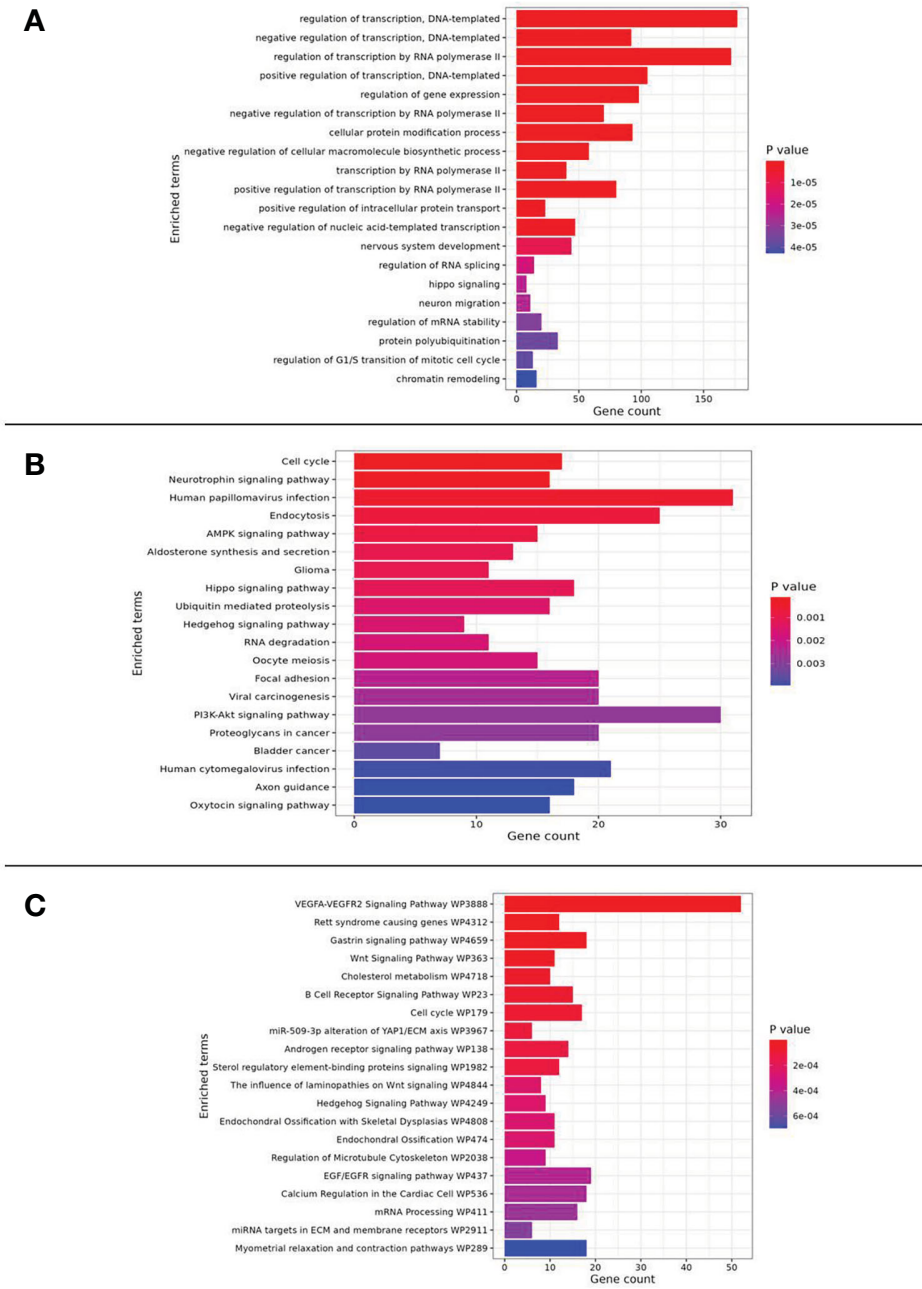
Pathway enrichment analysis results related to **(A)** GO_Biological Process, **(B)** KEGG and **(C)** WikiPathway databases.

Moreover, noteworthy is the correlation of miRNAs with the inhibition of the transient receptor potential melastatin 2 ion channel (TRPM2, also known as clusterin) and with Hippo pathway (**Figure 3**).

### DEmiRNAs in dietary regimen and relationship with metabolic features

**Figure 4:**
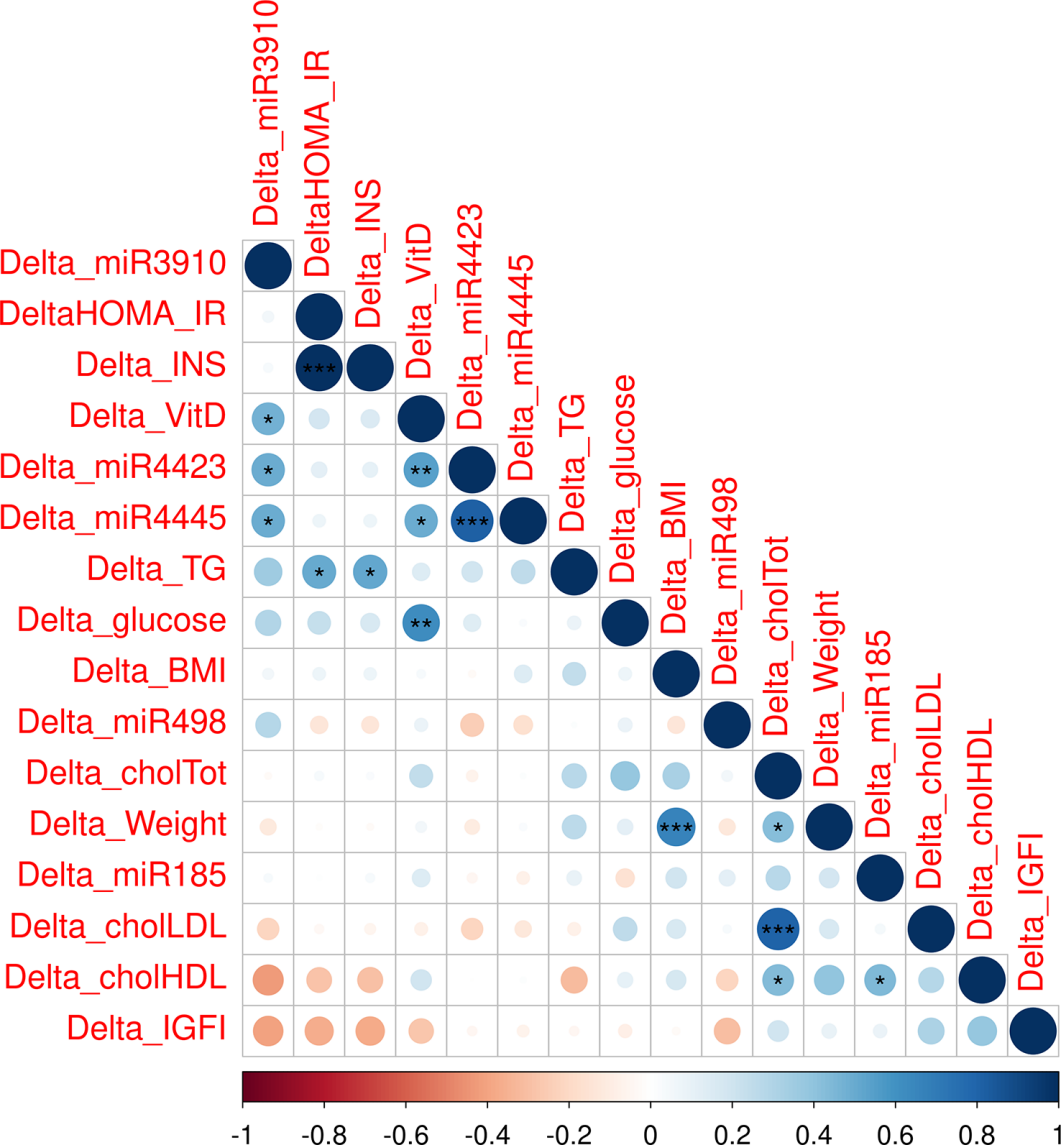
Correlation plot including differences between post and pre values of metabolic parameters and DEmiRNAs (INS: insulin; chol=cholesterol; TG: triglycerides; *, p-value< 0.05; **, p-value< 0.01; ***, p-value< 0.001).

To dissect the role of the validated DEmiRNAs, the variations in their expression levels, following the dietary intervention, were related to metabolic, and in particular, insulin-resistance parameters. Correlation plot in **Figure 4** displays significant relationships: ΔmiR-4423, ΔmiR-4445 and ΔmiR-3910 expressions are positively correlated with ΔvitaminD level; whilst ΔmiR-185-5p is related to ΔHDL cholesterol (**Figure 4**). The other metabolic parameters considered were unaffected by miRNAs deregulation.

**Figure 5:**
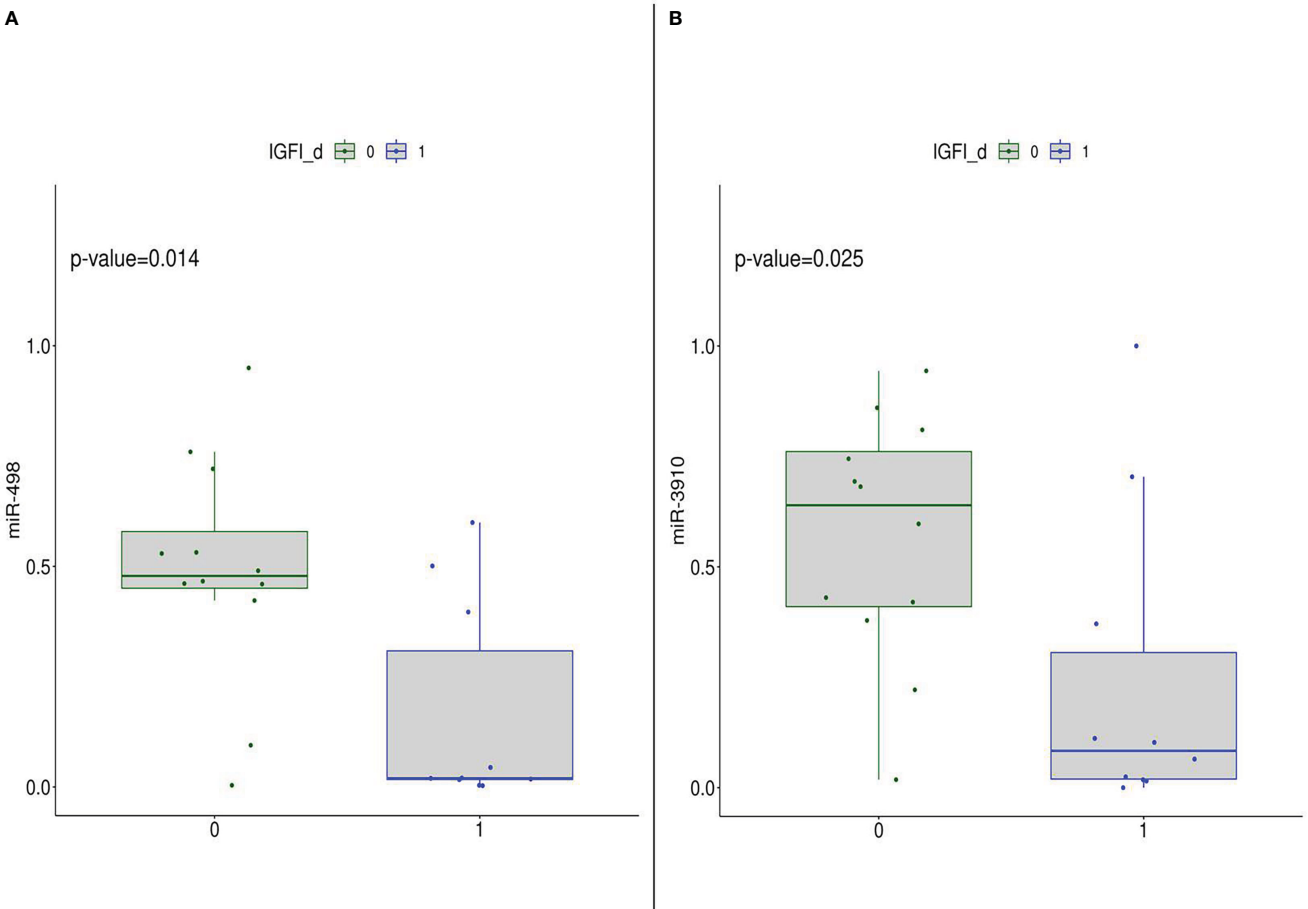
DEmiRNA (in detail, miR-498 **(A)** and miR-3910 **(B)**) expression in the post-diet group stratified according to IGF-I median levels, thus 0: subjects with IGF-I.≤median value; 1: subjects with IGF-I>median value.

Furthermore, focusing on the post-intervention group, the subset was stratified according to median values of metabolic parameters. Mann-Whithney test results highlighted that subjects with IGF-I levels lower than median value cut-off have significantly higher expression of miR-498 and miR-3910 than the other group (**Figure 5**). Such a result indicated that the diet intervention was able to modulate miR-498 and miR-3910 which *bona fide* are related to IGF-I parameter.

## Discussion

Disentangling the mechanisms necessary to improve diagnosis and prognosis of BC patients is a critical point since this is still a highly heterogeneous disease. The necessity to have a non-invasive biomarker for the disease monitoring must consider that circulating biomarkers may originate from many cells or tissues in the body, and in response to different conditions both physiological and pathological. Previous studies demonstrated the feasibility of using circulating miRNAs as biomarkers of tumors early diagnosis and prognosis (24).

In addition to the conventional approaches, a lifestyle intervention seems to be a good starting point to improve care of women affected by BC. In order to select peculiar circulating markers associated with a lifestyle intervention such as a diet in subjects with BRCA pathogenic alterations, we followed plasmatic miRNAs expression level after a Mediterranean dietary intervention in women carriers of BRCA mutations.

In our hands, plasma-circulating miR-185-5p, miR-498, miR-3910, miR-4423 and, miR-4445 were significantly up-regulated after the dietary intervention in the IG compared with their baseline levels and with the CG in the same conditions. These miRNAs are associated with crucial pathways in BC and may explain the mechanisms by which a Mediterranean dietary intervention affects breast cancer progression in BRCA-mutated women.

In the present study, a tumor-suppressor miRNA (25), miR-185-5p, often down-regulated in BC samples (26), was upregulated after the Mediterranean dietary intervention compared to the baseline levels but not in the CG (*P-value*<0.02) either in the training and in the validation set.

miR-185 is reported to be linked to the tumor size, the tumor stage, and the lymph node metastasis (26). In addition, a low miR-185 expression seems to contribute to the acquisition of stemness characteristics in BC cells (27). In detail, miR-185 targeted the cadherin 1 (CDH1) gene, which encodes for E-cadherin (E-cad) protein involved in the maintenance of pluripotency and self-renewal of embryonic stem cells and neural stem cells (28–30). Recently it has been reported that E-cad may have oncogenic properties in some tumor types. In particular, in BC cells a high E-cad expression level seems to enhance the metastasis formation (31).

The role in cancer of the other upregulated miRNAs after dietary intervention is still not well elucidated. Only miR-498 has been reported in literature to be often down-regulated in several tumor types such as colon and ovarian cancers (32, 33) and, also in BC (34). Furthermore, Leivonen et al. (2013) identified several miRNAs, among which miR-498 (35), regulating the human epidermal growth factor receptor 2 (ErbB2/HER2) pathway which is one of the driver pathway of BC, showing HER2 overexpression with the constitutive activation of its receptor and leading to a more aggressive form of the disease with the promotion of the metastatic process (35).

Pathway enrichment analysis performed using the five upregulated miRNAs (miR-185-5p, miR-498, miR-3910, miR-4423 and, miR-4445) and their target genes showed these miRNAs involvement in important interconnected pathways: the Hippo pathway, the PI3K-Akt, and the AMPK.

The Hippo pathway, a regulator in several signaling processes, such as organ size, tissue homeostasis, cell proliferation and differentiation, plays a peculiar role in BC (36–38), where it is reported an overstimulation of the AKT kinase activity, also *via* the HER2 protein, resulting in the majority of cases in a higher-grade carcinoma (38, 39). In particular, the Hippo pathway is a kinase cascade that results in the phosphorylation of the transcription co-activators YAP (yes-associated protein/yes-related protein) and TAZ (Tafazzin protein), inhibiting the activation of other transcription factors and, thus the tumor progression (40).

The AMPK activity is a serine/threonine kinase, mainly involved in the maintenance of cellular energy homeostasis by acting on glucose and lipid metabolism. Activated AMPK can lead to the stimulation of several regulatory processes involved in cancer pathogenesis (41). Recent studies reported AMPK as a novel druggable target (41).

These biological processes also involved specific metabolites controllable with the food intake. For instance, a link between the Hippo pathway and IGF-I is shown, since this molecule could modulate the hypoxia-activated YAP signaling, as previously demonstrated by Zhu et al. (2018) (42) and later confirmed also in BC by Rigiracciolo et al. (2020) (43). Furthermore, it has been highlighted how their inhibition could significantly enhance the efficacy of therapies.

Our work focused on the interactions between genetic profiles, lifestyle risk factors, and intermediate biomarkers. The inhibition of this complex network seems to be a requirement to develop anti-tumor strategies and we corroborate the hypothesis that miRNAs could improve the hormonal and metabolic pattern associated with BC risk, such as IGF-I, in response to the dietary intervention, slowing or inhibiting cancer growth in BRCA mutation carriers.

It has been reported that IGF-I is overexpressed in about 70% of BC patients and associated with higher BC risk (44), and this results in the activation of specific signaling pathways, i.e. Ras, Raf and mitogen-activated protein kinase (MAPK) and, the above-mentioned, PI3K-Akt and Hippo pathway. As previously discussed, BRCA1 negatively regulates IGF-I, and its defectiveness keeps always active the IGF-I/PI3K-Akt pathway, which significantly promotes tumor cell survival and proliferation (45–47). Hence, the carriers of BRCA mutation may be more affected by the mitogenic effect of insulin, stimulating mitosis and inhibiting apoptosis (48).

IGF-I could also, by the stimulation of different biological pathways, lead to significant TRPM2 expression (49).

The TRPM2 is a secreted glycoprotein that shows a key role in cell survival, cancer progression and drug resistance development (50). Its expression level is highly connected with various cellular stress responses (51); in fact, it has been reported overexpressed in several tumor types, including BC, involved in the promotion of cancer cell survival (52).

Several studies report that TRPM2 pharmacological inhibition could lead to a decreased tumor proliferation and viability, enhancing cell death and increasing drug sensitivity (53–55).

All these findings may corroborate the hypothesis to develop new drugs, starting from miRNAs as inhibitory molecules for these connected processes.

Moreover, as previously discussed, insulin resistance and obesity are linked with hereditary BC (11, 56).

We also reported the correlation of selected miRNAs with the levels of several molecules involved in metabolism in BC patients, such as vitamin D and cholesterol, creating a close connection between food, biological processes, metabolites and analyzed miRNAs.

Numerous studies, in fact, reported that low levels of vitamin D are linked to several pathological conditions, such as inflammation and cancer (57). Moreover, BC incidence and vitamin D levels seem to be negatively related (58), and this situation is associated with a worst outcomes for BC patients since this molecule is involved in the EMT process, inhibiting tumor migration, invasion, and metastatic potential (57, 59–62).

We observed that the changing in miRNA circulating levels in subjects that followed the dietary intervention is positively related to an increase in vitamin D levels in comparison to their baseline, contrary to what was shown in the CG, fortifying our thesis that a healthy diet, such as the Mediterranean diet, by influencing several biological processes, may represent an adjuvant approach to improve BC prevention and treatment.

Moreover, several studies focused their attention on the relationship between BC progression and cholesterol circulating levels. In particular, it has been reported that low levels of High-Density Lipoprotein (HDL) were associated with worse overall survival and disease free survival (63). Cancer cells, in fact, display a deregulated lipid metabolism, which can affect several cellular processes, including proliferation, differentiation and inflammation-related pathways.

In our hand we demonstrated that subjects who followed a specific sustenance, with the up-regulation of target miRNAs, displayed low levels of HDL compared to the CG. In addition, HDL-cholesterol levels have been reported to have a negative relationship with IGF-I expression (44).

In conclusion, we showed that post a short lifestyle intervention a significant increase of HDL circulating levels, but also a decrease in IGF-I and insulin expression were reported linked by miRNAs overexpression.

Therefore, these results, although further investigation are necessary, suggest that by changing the dietary habits of women at high genetic risk to develop BC, it is possible to affect different signaling pathways linked to cancer risk.

## Conclusion

Many studies have reported the benefits following a balanced diet to reduce the risk of multiple diseases, including BC. Here, we have widely discussed benefits to carry out lifestyle modification in subjects BRCA-mutated, who have a very high risk to develop BC, suggesting this approach as a risk-reduction measure.

Importantly, we highlighted the synergistic effect of a healthy diet and epigenetic regulation in BC through the modulation of specific miRNAs. Different miRNAs have been reported involved in the tumor onset acting as tumor suppressors by targeting tumor-associated genes that are often downregulated.

Accordingly, we showed that this situation could be reversed, albeit with a short period of dietary intervention, leading to a better physical condition of BRCA-mutated carriers.

Further investigations are necessary to elucidate if Mediterranean diet could really improve overall survival and disease-free survival in BC in a larger cohort, to develop primary prevention recommendations for women with BRCA mutations and provide an effective alternative therapeutic approach for this aggressive disease.

## Data availability statement

The datasets presented in this study can be found in online repositories. The names of the repository/repositories and accession number(s) can be found below: https://www.ncbi.nlm.nih.gov/, GSE223101.

## Ethics statement

The studies involving human participants were reviewed and approved by Comitato Etico IRCCS Istituto Tumori “Giovanni Paolo II” “Gabriella Serio”. The patients/participants provided their written informed consent to participate in this study.

## Author contributions

SD performed bioinformatic and statistical analyses and wrote sections of the manuscript. DT set up all validation experiments. ST and PP contributed to conception and design of the study. AD, AT and KD contributed to the cohort enrollment and collection of data. DT, AO, EB, MD contributed to write the draft of the manuscript. All authors contributed to the article and approved the submitted version.

## References

[1] Allahyari E Velaei K Sanaat Z Jalilzadeh N Mehdizadeh A Rahmati M. RNA Interference: Promising approach for breast cancer diagnosis and treatment. Cell Biol Int (2022). doi: 10.1002/cbin.11979 36571107

[2] Sun X Liu K Lu S He W Du Z. Targeted therapy and immunotherapy for heterogeneous breast cancer. Cancers (Basel) (2022) 14(21):5456. doi: 10.3390/cancers14215456 36358874 PMC9656512

[3] Kwon Y-J Cho Y-E Cho A-R Choi WJ Yun S Park H-. The possible influence of Mediterranean diet on extracellular vesicle miRNA expression in breast cancer survivors. Cancers (Basel) (2020) 12. doi: 10.3390/cancers12061355 PMC7352167 32466456

[4] Burke S Wurz A Bradshaw A Saunders S West MA Brunet J. Physical activity and quality of life in cancer survivors: A meta-synthesis of qualitative research. Cancers (Basel) (2017) 9. doi: 10.3390/cancers9050053 PMC5447963 28531109

[5] Playdon MC Bracken MB Sanft TB Ligibel JA Harrigan M Irwin ML. Weight gain after breast cancer diagnosis and all-cause mortality: Systematic review and meta-analysis. J Natl Cancer Inst (2015) 107:djv275. doi: 10.1093/jnci/djv275 26424778 PMC4715249

[6] Adams BD Arem H Hubal MJ Cartmel B Li F Harrigan M. Exercise and weight loss interventions and miRNA expression in women with breast cancer. Breast Cancer Res Treat (2018) 170:55–67. doi: 10.1007/s10549-018-4738-6 29511965 PMC6444907

[7] Mota de Sá P Richard AJ Hang H Stephens JM. Transcriptional regulation of adipogenesis. Compr Physiol (2017) 7:635–74. doi: 10.1002/cphy.c160022 28333384

[8] Spiegelman BM Flier JS. Adipogenesis and obesity: Rounding out the big picture. Cell (1996) 87:377–89. doi: 10.1016/s0092-8674(00)81359-8 8898192

[9] Oliverio A Radice P Colombo M Paradiso A Tommasi S Daniele A. The impact of Mediterranean dietary intervention on metabolic and hormonal parameters according to BRCA1/2 variant type. Front Genet (2022) 13:820878. doi: 10.3389/fgene.2022.820878 35356420 PMC8959623

[10] Norat T Dossus L Rinaldi S Overvad K Grønbaek H Tjønneland A. Diet, serum insulin-like growth factor-I and IGF-binding protein-3 in European women. Eur J Clin Nutr (2007) 61:91–8. doi: 10.1038/sj.ejcn.1602494 16900085

[11] Bordeleau L Lipscombe L Lubinski J Ghadirian P Foulkes WD Neuhausen S. Diabetes and breast cancer among women with BRCA1 and BRCA2 mutations. Cancer (2011) 117:1812–8. doi: 10.1002/cncr.25595 PMC3413077 21509758

[12] Pasanisi P Bruno E Venturelli E Manoukian S Barile M Peissel B. Serum levels of IGF-I and BRCA penetrance: a case control study in breast cancer families. Fam Cancer (2011) 10:521–8. doi: 10.1007/s10689-011-9437-y 21455766

[13] Angelescu MA Andronic O Dima SO Popescu I Meivar-Levy I Ferber S. miRNAs as biomarkers in diabetes: Moving towards precision medicine. Int J Mol Sci (2022) 23. doi: 10.3390/ijms232112843 PMC9655971 36361633

[14] Daniele A Divella R Pilato B Tommasi S Pasanisi P Patruno M. Can harmful lifestyle, obesity and weight changes increase the risk of breast cancer in BRCA 1 and BRCA 2 mutation carriers? a mini review. Hered Cancer Clin Pract (2021) 19:45. doi: 10.1186/s13053-021-00199-6 34706754 PMC8554866

[15] Wan D Li V Banfield L Azab S de Souza RJ Anand SS. Diet and nutrition in peripheral artery disease: A systematic review. Can J Cardiol (2022) 38:672–80. doi: 10.1016/j.cjca.2022.01.021 35307328

[16] Tang X Tang G Ozcan S. Role of microRNAs in diabetes. Biochim Biophys Acta (2008) 1779:697–701. doi: 10.1016/j.bbagrm.2008.06.010 18655850 PMC2643014

[17] Falzone L Grimaldi M Celentano E Augustin LSA Libra M. Identification of modulated MicroRNAs associated with breast cancer, diet, and physical activity. Cancers (Basel) (2020) 12. doi: 10.3390/cancers12092555 PMC7564431 32911851

[18] Jin Y Wong YS Goh BKP Chan CY Cheow PC Chow PKH. Circulating microRNAs as potential diagnostic and prognostic biomarkers in hepatocellular carcinoma. Sci Rep (2019) 9:10464. doi: 10.1038/s41598-019-46872-8 31320713 PMC6639394

[19] Bruno E Oliverio A Paradiso AV Daniele A Tommasi S Tufaro A. A Mediterranean dietary intervention in female carriers of BRCA mutations: Results from an Italian prospective randomized controlled trial. Cancers (Basel) (2020) 12. doi: 10.3390/cancers12123732 PMC7764681 33322597

[20] Bruno E Manoukian S Venturelli E Oliverio A Rovera F Iula G. Adherence to Mediterranean diet and metabolic syndrome in BRCA mutation carriers. Integr Cancer Ther (2018) 17:153–60. doi: 10.1177/1534735417721015 PMC5950953 28741383

[21] Pasanisi P Bruno E Venturelli E Morelli D Oliverio A Baldassari I. A dietary intervention to lower serum levels of IGF-I in BRCA mutation carriers. Cancers (Basel) (2018) 10. doi: 10.3390/cancers10090309 PMC6162406 30181513

[22] Durnin JV Womersley J. Body fat assessed from total body density and its estimation from skinfold thickness: measurements on 481 men and women aged from 16 to 72 years. Br J Nutr (1974) 32:77–97. doi: 10.1079/bjn19740060 4843734

[23] Karagkouni D Paraskevopoulou MD Chatzopoulos S Vlachos IS Tastsoglou S Kanellos I. DIANA-TarBase v8: a decade-long collection of experimentally supported miRNA-gene interactions. Nucleic Acids Res (2018) 46:D239–45. doi: 10.1093/nar/gkx1141 PMC5753203 29156006

[24] Yao Y Liu R Gao C Zhang T Qi L Liu G. Identification of prognostic biomarkers for breast cancer based on miRNA and mRNA co-expression network. J Cell Biochem (2019) 120:15378–88. doi: 10.1002/jcb.28805 31037764

[25] Ma X Shen D Li H Zhang Y Lv X Huang Q. MicroRNA-185 inhibits cell proliferation and induces cell apoptosis by targeting VEGFA directly in von hippel-lindau-inactivated clear cell renal cell carcinoma. Urol Oncol (2015) 33:169.e1–11. doi: 10.1016/j.urolonc.2015.01.003 25700976

[26] Shahabi A Naghili B Ansarin K Montazeri M Dadashpour M Zarghami N. Let-7d and miR-185 impede epithelial-mesenchymal transition by downregulating Rab25 in breast cancer. Asian Pac J Cancer Prev (2021) 22:305–13. doi: 10.31557/APJCP.2021.22.1.305 PMC8184182 33507713

[27] Lu G Li Y Ma Y Lu J Chen Y Jiang Q. Long noncoding RNA LINC00511 contributes to breast cancer tumourigenesis and stemness by inducing the miR-185-3p/E2F1/Nanog axis. J Exp Clin Cancer Res (2018) 37:289. doi: 10.1186/s13046-018-0945-6 30482236 PMC6260744

[28] Soncin F Ward CM. The function of e-cadherin in stem cell pluripotency and self-renewal. Genes (Basel) (2011) 2:229–59. doi: 10.3390/genes2010229 PMC3924836 24710147

[29] Karpowicz P Willaime-Morawek S Balenci L DeVeale B Inoue T van der Kooy D. E-cadherin regulates neural stem cell self-renewal. J Neurosci Off J Soc Neurosci (2009) 29:3885–96. doi: 10.1523/JNEUROSCI.0037-09.2009 PMC6665048 19321785

[30] Redmer T Diecke S Grigoryan T Quiroga-Negreira A Birchmeier W Besser D. E-cadherin is crucial for embryonic stem cell pluripotency and can replace OCT4 during somatic cell reprogramming. EMBO Rep (2011) 12:720–6. doi: 10.1038/embor.2011.88 PMC3128971 21617704

[31] Padmanaban V Krol I Suhail Y Szczerba BM Aceto N Bader JS. E-cadherin is required for metastasis in multiple models of breast cancer. Nature (2019) 573:439–44. doi: 10.1038/s41586-019-1526-3 PMC7365572 31485072

[32] Gopalan V Smith RA Lam AK-Y. Downregulation of microRNA-498 in colorectal cancers and its cellular effects. Exp Cell Res (2015) 330:423–8. doi: 10.1016/j.yexcr.2014.08.006 25128149

[33] Kasiappan R Shen Z Tse AK-W Jinwal U Tang J Lungchukiet P. 1,25-dihydroxyvitamin D3 suppresses telomerase expression and human cancer growth through microRNA-498. J Biol Chem (2012) 287:41297–309. doi: 10.1074/jbc.M112.407189 PMC3510828 23055531

[34] Matamala N Vargas MT González-Cámpora R Arias JI Menéndez P Andrés-León E. MicroRNA deregulation in triple negative breast cancer reveals a role of miR-498 in regulating BRCA1 expression. Oncotarget (2016) 7:20068–79. doi: 10.18632/oncotarget.7705 PMC4991439 26933805

[35] Leivonen S-K Sahlberg KK Mäkelä R Due EU Kallioniemi O Børresen-Dale A-L. High-throughput screens identify microRNAs essential for HER2 positive breast cancer cell growth. Mol Oncol (2014) 8:93–104. doi: 10.1016/j.molonc.2013.10.001 24148764 PMC5528509

[36] Yousefi H Delavar MR Piroozian F Baghi M Nguyen K Cheng T. Hippo signaling pathway: A comprehensive gene expression profile analysis in breast cancer. BioMed Pharmacother (2022) 151:113144. doi: 10.1016/j.biopha.2022.113144 35623167

[37] Li F-L Guan K-L. The two sides of hippo pathway in cancer. Semin Cancer Biol (2022) 85:33–42. doi: 10.1016/j.semcancer.2021.07.006 34265423

[38] He L Liu X Yang J Li W Liu S Liu X. Imbalance of the reciprocally inhibitory loop between the ubiquitin-specific protease USP43 and EGFR/PI3K/AKT drives breast carcinogenesis. Cell Res (2018) 28:934–51. doi: 10.1038/s41422-018-0079-6 PMC6123467 30135474

[39] Sun M Wang G Paciga JE Feldman RI Yuan ZQ Ma XL. AKT1/PKBalpha kinase is frequently elevated in human cancers and its constitutive activation is required for oncogenic transformation in NIH3T3 cells. Am J Pathol (2001) 159:431–7. doi: 10.1016/s0002-9440(10)61714-2 PMC1850562 11485901

[40] Pan D. The hippo signaling pathway in development and cancer. Dev Cell (2010) 19:491–505. doi: 10.1016/j.devcel.2010.09.011 20951342 PMC3124840

[41] Li W Saud SM Young MR Chen G Hua B. Targeting AMPK for cancer prevention and treatment. Oncotarget (2015) 6:7365–78. doi: 10.18632/oncotarget.3629 PMC4480686 25812084

[42] Zhu H Wang D-D Yuan T Yan F-J Zeng C-M Dai X-Y. Multikinase inhibitor CT-707 targets liver cancer by interrupting the hypoxia-activated IGF-1R-YAP axis. Cancer Res (2018) 78:3995–4006. doi: 10.1158/0008-5472.CAN-17-1548 29669759

[43] Rigiracciolo DC Nohata N Lappano R Cirillo F Talia M Scordamaglia D. IGF-1/IGF-1R/FAK/YAP transduction signaling prompts growth effects in triple-negative breast cancer (TNBC) cells. Cells (2020) 9. doi: 10.3390/cells9041010 PMC7225986 32325700

[44] Schernhammer ES Holly JM Pollak MN Hankinson SE. Circulating levels of insulin-like growth factors, their binding proteins, and breast cancer risk. Cancer Epidemiol Biomarkers Prev (2005) 14:699–704. doi: 10.1158/1055-9965.EPI-04-0561 15767352

[45] Kang HJ Yi YW Kim HJ Hong YB Seong YS Bae I. BRCA1 negatively regulates IGF-1 expression through an estrogen-responsive element-like site. Cell Death Dis (2012) 3:e336. doi: 10.1038/cddis.2012.78 22739988 PMC3388245

[46] Nielsen TO Andrews HN Cheang M Kucab JE Hsu FD Ragaz J. Expression of the insulin-like growth factor I receptor and urokinase plasminogen activator in breast cancer is associated with poor survival: potential for intervention with 17-allylamino geldanamycin. Cancer Res (2004) 64:286–91. doi: 10.1158/0008-5472.can-03-1242 14729636

[47] Elumalai P Arunkumar R Benson CS Sharmila G Arunakaran J. Nimbolide inhibits IGF-i-mediated PI3K/Akt and MAPK signalling in human breast cancer cell lines (MCF-7 and MDA-MB-231). Cell Biochem Funct (2014) 32:476–84. doi: 10.1002/cbf.3040 24888707

[48] Draznin B. Mitogenic action of insulin: friend, foe or “frenemy”? Diabetologia (2010) 53:229–33. doi: 10.1007/s00125-009-1558-6 19851749

[49] Luo X Suzuki M Ghandhi SA Amundson SA Boothman DA. ATM Regulates insulin-like growth factor 1-secretory clusterin (IGF-1-sCLU) expression that protects cells against senescence. PloS One (2014) 9:e99983. doi: 10.1371/journal.pone.0099983 24937130 PMC4061041

[50] Yang P Yang Z Dong Y Yang L Peng S Yuan L. Clusterin is a biomarker of breast cancer prognosis and correlated with immune microenvironment. Transl Cancer Res (2023) 12:31–45. doi: 10.21037/tcr-22-1882 36760385 PMC9906057

[51] Jo H Jia Y Subramanian KK Hattori H Luo HR. Cancer cell-derived clusterin modulates the phosphatidylinositol 3’-kinase-Akt pathway through attenuation of insulin-like growth factor 1 during serum deprivation. Mol Cell Biol (2008) 28:4285–99. doi: 10.1128/MCB.01240-07 PMC2447147 18458059

[52] Miller BA. TRPM2 in cancer. Cell Calcium (2019) 80:8–17. doi: 10.1016/j.ceca.2019.03.002 30925291 PMC6545160

[53] Orfanelli U Wenke A-K Doglioni C Russo V Bosserhoff AK Lavorgna G. Identification of novel sense and antisense transcription at the TRPM2 locus in cancer. Cell Res (2008) 18:1128–40. doi: 10.1038/cr.2008.296 18957938

[54] Koh DW Powell DP Blake SD Hoffman JL Hopkins MM Feng X. Enhanced cytotoxicity in triple-negative and estrogen receptor−positive breast adenocarcinoma cells due to inhibition of the transient receptor potential melastatin-2 channel. Oncol Rep (2015) 34:1589–98. doi: 10.3892/or.2015.4131 PMC4735697 26178079

[55] Almasi S Kennedy BE El-Aghil M Sterea AM Gujar S Partida-Sánchez S. TRPM2 channel-mediated regulation of autophagy maintains mitochondrial function and promotes gastric cancer cell survival *via* the JNK-signaling pathway. J Biol Chem (2018) 293:3637–50. doi: 10.1074/jbc.M117.817635 PMC5846146 29343514

[56] Dumais V Lumingu J Bedard M Paquet L Verma S Fontaine-Bisson B. Prevalence of insulin resistance, metabolic syndrome, and type 2 diabetes in Canadian women at high risk for breast cancer. Breast J (2017) 23:482–3. doi: 10.1111/tbj.12772 28121048

[57] Mego M Vlkova B Minarik G Cierna Z Karaba M Benca J. Vitamin d and circulating tumor cells in primary breast cancer. Front Oncol (2022) 12:950451. doi: 10.3389/fonc.2022.950451 36158648 PMC9489852

[58] Chen P Hu P Xie D Qin Y Wang F Wang H. Meta-analysis of vitamin d, calcium and the prevention of breast cancer. Breast Cancer Res Treat (2010) 121:469–77. doi: 10.1007/s10549-009-0593-9 19851861

[59] Goodwin PJ Ennis M Pritchard KI Koo J Hood N. Prognostic effects of 25-hydroxyvitamin d levels in early breast cancer. J Clin Oncol Off J Am Soc Clin Oncol (2009) 27:3757–63. doi: 10.1200/JCO.2008.20.0725 19451439

[60] Freedman DM Looker AC Chang S-C Graubard BI. Prospective study of serum vitamin d and cancer mortality in the united states. J Natl Cancer Inst (2007) 99:1594–602. doi: 10.1093/jnci/djm204 17971526

[61] Manousaki D Richards JB. Low vitamin d levels as a risk factor for cancer. BMJ (2017) 359:j4952. doi: 10.1136/bmj.j4952 29089329

[62] Peppone LJ Rickles AS Janelsins MC Insalaco MR Skinner KA. The association between breast cancer prognostic indicators and serum 25-OH vitamin d levels. Ann Surg Oncol (2012) 19:2590–9. doi: 10.1245/s10434-012-2297-3 PMC4158737 22446898

[63] Centonze G Natalini D Piccolantonio A Salemme V Morellato A Arina P. Cholesterol and its derivatives: Multifaceted players in breast cancer progression. Front Oncol (2022) 12:906670. doi: 10.3389/fonc.2022.906670 35719918 PMC9204587

