# Supplementary material for: miRNA deregulation and relationship with metabolic parameters after Mediterranean dietary intervention in BRCA-mutated women
Source: Front Oncol. 2023 Apr 4;13:1147190. doi: 10.3389/fonc.2023.1147190 (PMC10110888; doi:10.3389/fonc.2023.1147190)
Supplement: Supplementary file 1 [file Table_1.docx]

**Supplementary Table 1.** BRCA1 and BRCA2 mutations of the study population by treatment group.

|  | **Diet** | **Control** |
| --- | --- | --- |
| 236 | BRCA2: c.6468_6469delTC (rs80359596) |  |
| 237 |  | BRCA1: c.5266dupC (rs397507247) |
| 238 |  | BRCA1: c.5266dupC (rs397507247) |
| 239 | BRCA1: c.5266dupC (rs397507247) |  |
| 240 | BRCA1: c.5382insC stop1829 |  |
| 241 | BRCA1: c.5382insC stop1830 |  |
| 242 | BRCA1: c.5382insC stop1831 |  |
| 243 | BRCA1: c.5266dupC (rs397507247) |  |
| 247 | BRCA1: c.5266dupC (rs397507247) |  |
| 248 | BRCA1: c.5263_5264insC (rs80357906) |  |
| 249 | BRCA1: c.5266dupC (rs397507247) |  |
| 250 | BRCA1: c.4484G>T (rs80357389) |  |
| 251 |  | BRCA1: c.5266dupC (rs397507247) |
| 252 |  | BRCA1: c.798_799delTT (rs80357724) |
| 288 | BRCA2: c.5796_5797delTA (rs80359537) |  |
| 289 | BRCA2: c.5796_5797delTA (rs80359537) |  |
| 291 |  | BRCA2: c.2024del5 stop599 |
| 293 |  | BRCA2: c.2024del5 stop599 |
| 294 |  | BRCA1: c.5266dupC (rs397507247) |
| 295 |  | BRCA1: c.5266dupC (rs397507247) |
| 299 |  | BRCA1: c.5266dupC (rs397507247) |
| 301 |  | BRCA2: c.2024del5 stop599 |
| 303 |  | BRCA2: c.1794_1798delATCTT (rs276174813) |
| 364 |  | BRCA1: c.5266dupC (rs397507247) |
| 366 | BRCA1: c.5266dupC (rs397507247) |  |
| 373 |  | BRCA1: c.5266dupC (rs397507247) |
| 490 | BRCA1: c.1687C>T (rs80356898) |  |
| 497 | BRCA1: c.5266dupC (rs397507247) |  |
| 499 |  | BRCA2: c.67+1G>A (rs81002796) |
| 500 | BRCA1: c.5266dupC (rs397507247) |  |
| 298 | BRCA2: c.6468_6469delTC (rs80359597) |  |
| 302 | BRCA1: c.5266dupC (rs397507247) |  |
| 304 |  | BRCA2: c.1794_1798delATCTT (rs276174813) |
| 305 | BRCA1: c.5266dupC (rs397507247) |  |
| 306 | BRCA2: c.6468_6469delTC (rs80359597) |  |
| 363 |  | BRCA1: c.5266dupC (rs397507247) |
| 365 |  | BRCA1: c.5266dupC (rs397507247) |
| 367 | BRCA1: c.5266dupC (rs397507247) |  |
| 369 | BRCA2: c.5796_5797delTA (rs80359537) |  |
| 370 | BRCA2:c.2049_2050delTC (rs80359319) |  |
| 371 | BRCA2: c.2049_2050delTC (rs80359319) |  |
| 372 |  | BRCA1: c.5266dupC (rs397507247) |
| 374 |  | BRCA2: c.6468_6469delTC (rs80359597) |
| 375 | BRCA2: c.5796_5797delTA (rs80359537) |  |
| 409 |  | BRCA1: c.5266dupC (rs397507247) |
| 413 |  | BRCA2: c.2151T>A (rs876660512) |
| 415 |  | BRCA1: c.3228_3229del (rs80357635) |
| 422 |  | BRCA1: c.5266dupC (rs397507247) |
| 491 |  | BRCA2: c.2151T>A (rs876660512) |
| 492 | BRCA2: c.9007_9007delG |  |
| 493 |  | BRCA2: c.6468_6469delTC (rs80359597) |
| 495 | BRCA2: c.6468_6469delTC (rs80359597) |  |
| 496 |  | BRCA2: c.6468_6469delTC (rs80359597) |
| 502 |  | BRCA1: c.5266dupC (rs397507247) |
| 508 | BRCA1: c.5266dupC (rs397507247) |  |
| 510 | BRCA1: c.3228_3229del (rs80357635) |  |

**Supplementary Table 2.** Baseline characteristics of the study population by sample set.

| **Characteristic** | **Training cohort**, N = 30^1^ | **Validation cohort**, N = 26^1^ | **p-value**^2^ |
| --- | --- | --- | --- |
| Gene mutation (%) |  |  | 0.3 |
| *BRCA1* | 20 (67%) | 14 (54%) |  |
| *BRCA2* | 10 (33%) | 12 (46%) |  |
| Diet vs Control |  |  | 0.8 |
| Control | 14 (47%) | 13 (50%) |  |
| Diet | 16 (53%) | 13 (50%) |  |
| Age | 51 (38, 56) | 48 (43, 52) | 0.9 |
| Height (cm) | 163 (160, 166) | 161 (156, 164) | 0.040 |
| Fat Mass | 38.2 (33.8, 41.7) | 32.2 (30.0, 37.1) | 0.06 |
| Adiponectin (ng/ml) | 9.65 (6.39, 13.57) | 9.9 (6.27, 15.5) | 0.6 |
| Leptin (ng/ml) | 18 (13, 31) | 21 (12, 5.58) | 0.3 |
| Weight (kg) | 67 (58, 75) | 58 (52, 62) | 0.059 |
| BMI | 24.4 (21.0, 28.3) | 22.4 (20.4, 24.2) | 0.058 |
| Glycemia (ml/dl) | 91 (87, 96) | 89 (87, 93) | 0.2 |
| Cholesterol Tot (mg/dl) | 198 (163, 221) | 199 (188, 219) | 0.7 |
| Cholesterol HDL | 67 (54, 76) | 68 (61, 80) | 0.2 |
| Cholesterol LDL | 122 (86, 158) | 134 (97, 151) | 0.4 |
| Triglycerides (mg/dl) | 87 (71, 106) | 80 (64, 100) | 0.5 |
| Vitamin D (μg/L) | 28 (21, 36) | 24 (20, 32) | 0.5 |
| IGF-I (ng/ml) | 131 (117, 158) | 150 (123, 206) | 0.2 |
| Insulin (μU/ml) | 6 (5, 10) | 4 (3, 8) | 0.11 |
| HOMA1_IR | 1.38 (0.98, 2.27) | 0.95 (0.64, 1.79) | 0.10 |
| ^1^n (%); Median (IQR) | | | |
| ^2^Pearson's Chi-squared test; Wilcoxon rank sum test | | | |
